# Malaria positivity following a single oral dose of azithromycin among children in Burkina Faso: a randomized controlled trial

**DOI:** 10.1186/s12879-022-07296-4

**Published:** 2022-03-25

**Authors:** Jessica Brogdon, Clarisse Dah, Ali Sié, Mamadou Bountogo, Boubacar Coulibaly, Idrissa Kouanda, Mamadou Ouattara, Guillaume Compaoré, Eric Nebie, Mariam Seynou, Elodie Lebas, Fanice Nyatigo, Huiyu Hu, Benjamin F. Arnold, Thomas M. Lietman, Catherine E. Oldenburg

**Affiliations:** 1 Francis I Proctor Foundation, University of California, San Francisco, 490 Illinois Street, Floor 2, San Francisco, CA 94158 USA; 2 Centre de Recherche en Santé de Nouna, Nouna, Burkina Faso; 3 Department of Ophthalmology, University of California, San Francisco, San Francisco, CA USA; 4 Department of Epidemiology and Biostatistics, University of California, San Francisco, San Francisco, CA USA

**Keywords:** Azithromycin, Sahel, Malaria, Randomized controlled trial

## Abstract

**Background:**

Azithromycin is a broad-spectrum antibiotic that has moderate antimalarial activity and has been shown to reduce all-cause mortality when biannually administered to children under five in high mortality settings in sub-Saharan Africa. One potential mechanism for this observed reduction in mortality is via a reduction in malaria transmission.

**Methods:**

We evaluated whether a single oral dose of azithromycin reduces malaria positivity by rapid diagnostic test (RDT). We conducted an individually randomized placebo-controlled trial in Burkina Faso during the high malaria transmission season in August 2020. Children aged 8 days to 59 months old were randomized to a single oral dose of azithromycin (20 mg/kg) or matching placebo. At baseline and 14 days following treatment, we administered a rapid diagnostic test (RDT) to detect *Plasmodium falciparum* and measured tympanic temperature for all children. Caregiver-reported adverse events and clinic visits were recorded at the day 14 visit.

**Results:**

We enrolled 449 children with 221 randomized to azithromycin and 228 to placebo. The median age was 32 months and 48% were female. A total of 8% of children had a positive RDT for malaria at baseline and 11% had a fever (tympanic temperature ≥ 37.5 °C). In the azithromycin arm, 8% of children had a positive RDT for malaria at 14 days compared to 7% in the placebo arm (*P* = 0.65). Fifteen percent of children in the azithromycin arm had a fever ≥ 37.5 °C compared to 21% in the placebo arm (*P* = 0.12). Caregivers of children in the azithromycin group had lower odds of reporting fever as an adverse event compared to children in the placebo group (OR 0.41, 95% CI 0.18–0.96, *P* = 0.04). Caregiver-reported clinic visits were uncommon, and there were no observed differences between arms (*P* = 0.32).

**Conclusions:**

We did not find evidence that a single oral dose of azithromycin reduced malaria positivity during the high transmission season. Caregiver-reported fever occurred less often in children receiving azithromycin compared to placebo, indicating that azithromycin may have some effect on non-malarial infections.

*Trial registration* Clinicaltrials.gov NCT04315272, registered 19/03/2020

## Introduction

Children under 5 are the most vulnerable age group affected by malaria, accounting for 67% of all malaria deaths in 2019 [1]. The malaria burden is often greatest during the first few years of life, before natural immunity is acquired [2]. Interventions such as seasonal malaria chemoprevention (SMC) have reduced deaths from severe malaria, but growing resistance to first line antimalarial drugs threatens to impede progress [3, 4]. Azithromycin is a macrolide with modest anti-malarial properties that has been shown to have a negative impact on the asexual stages of the *Plasmodium falciparum* parasite [5]. While azithromycin is not widely used for malaria control, mass biannual azithromycin distribution has been shown to reduce all-cause child mortality and malaria parasitemia among preschool aged children in some settings [6–8].

Burkina Faso is hyperendemic for malaria with more than 20 million people at risk [9]. The Burkinabè Ministry of Health estimates that 66% of all deaths in children under 5 were attributable to malaria in 2018 [10]. Here we investigate changes in malaria positivity determined by rapid diagnostic test (RDT) among Burkinabè children 8 days to 59 months old who were individually randomized to receive a single dose of azithromycin or placebo. We hypothesized that children receiving azithromycin would have lower RDT positivity after a 14-day period compared to those receiving placebo.

## Methods

### Study overview

This study was a placebo-controlled individually randomized trial evaluating a single oral dose of azithromycin (20 mg/kg) compared to placebo for malaria among children under 5 (ClinicalTrials.gov NCT04315272, registered 19/03/2020). We conducted assessments at baseline and 14 days following enrollment. We also collected stool samples from the participants over a 6-month period, but these results will be reported separately. The trial was approved by the Comité National d’Ethique pour la Recherche (National Ethics Committee of Burkina Faso) in Ouagadougou, Burkina Faso and the Institutional Review Board at the University of California, San Francisco. Written informed consent was obtained from the caregiver prior to enrollment.

### Study setting

**Fig. 1:**
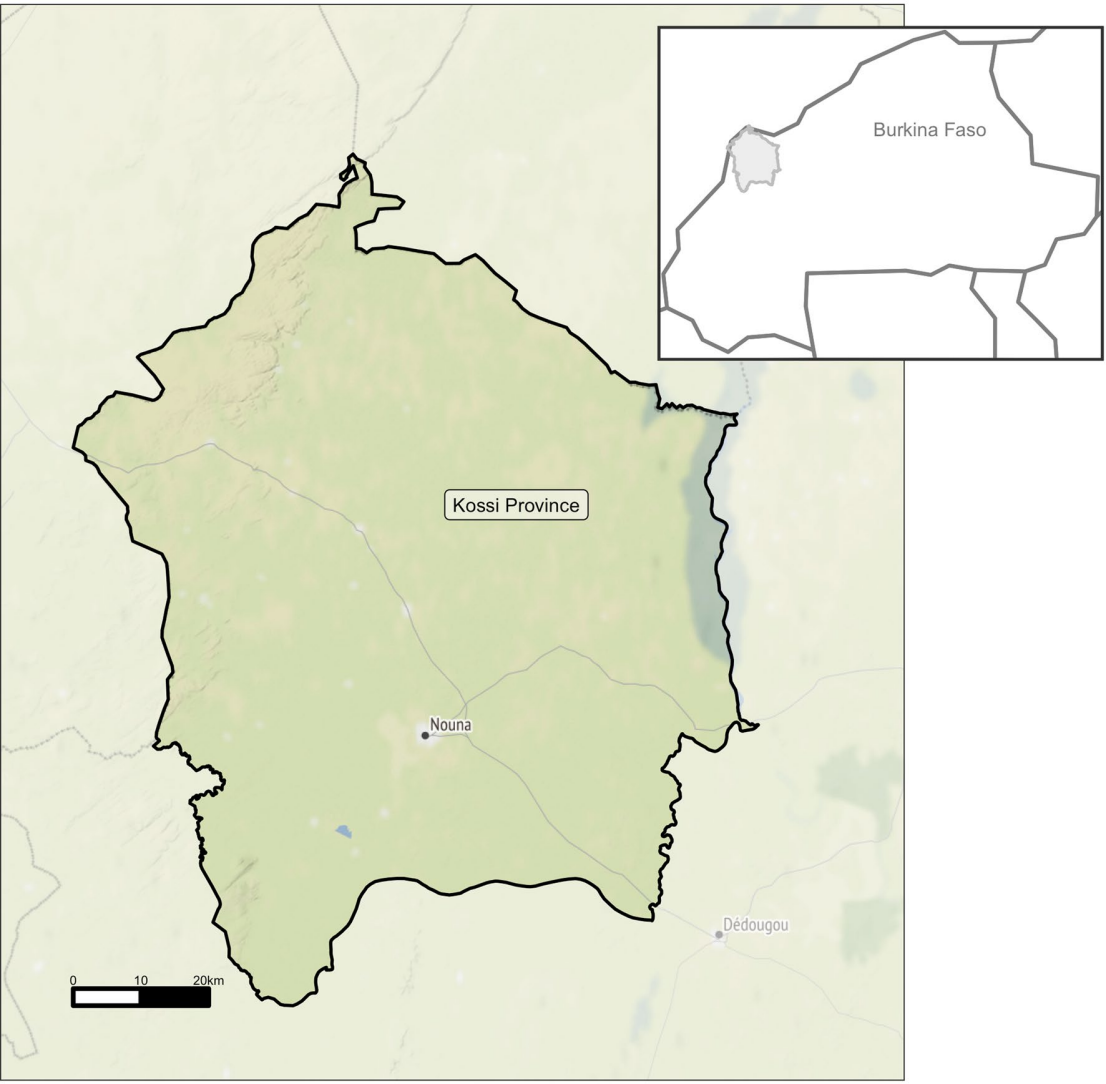
Map of the study area

The trial was conducted in Nouna town in northwestern Burkina Faso, which is approximately 300 km from the capital city Ouagadougou. Nouna is the capital of Kossi province with an estimated 25,000 inhabitants of various ethnic groups (Fig. 1) [11]. The population is peri-urban and consists almost exclusively of subsistence farmers. The climate is sub-Saharan, with an estimated mean annual rainfall of 796 mm [12]. The Nouna Health Research Center (CRSN) is a partner of the Ministry of Health and implements the Nouna Health and Demographic Surveillance System (HDSS). The HDSS encompasses the entire Nouna district including approximately 59 villages and 107,000 inhabitants. The HDSS was established in 1992 for the collection of longitudinal data to assess natality, mortality, and migration [12–14].

Children were enrolled in mid-August 2020 at the Nouna District Hospital. The primary endpoint occurred 2 weeks following enrollment in September 2020. In this setting malaria transmission is highly seasonal and typically peaks from July through October during the rainy season [15]. Seasonal malaria chemoprophylaxis was administered concurrently to children aged 3–59 months on a monthly basis from July to October in Nouna town. The predominant malaria vector in the region is the *Anopheles gambiae* complex, and the *Anopheles coluzzi* species is most commonly found in Nouna [16]. *P. falciparum* is the primary malaria species infecting humans in the region [17].

### Recruitment and eligibility

Mobilizers sensitized the community by visiting households with children under 5 based on the most recent census conducted by the Nouna Health and Demographic Surveillance Site (HDSS) [14]. Study staff informed caregivers about the study, and interested participants were encouraged to present to the Nouna District Hospital to be assessed for eligibility. Children meeting the following criteria were eligible for the study: age between 8 days and 59 months old, primary residence within Nouna town, available for the next 6-month period, no known allergy to macrolides, and able to orally feed (to swallow the study medication).

### Baseline assessment

At baseline, study staff conducted a survey with the caregiver. Questions included breastfeeding status, maternal age, the mother’s level of education, literacy, and gravidity. The study staff member utilized a custom mobile application to input all data into a handheld tablet (Dimagi, Inc., CommCare, 2020).

### Malaria assessment

An *OnSite* Pf/Pan antigen rapid test (CTK Biotech Inc, USA) was used to detect *Plasmodium falciparum* among all participants regardless of presence of fever at baseline and 14 days following treatment. Study staff measured tympanic temperature for all children at both study visits using the Braun Thermoscan 7 Digital Ear thermometer (Kaz, Inc., USA). Fever was defined as tympanic temperature ≥ 37.5 °C. Febrile children and those with a positive malaria RDT were referred for care.

### Intervention

Enrolled participants were randomized to receive a single oral dose of azithromycin (20 mg/kg) or equivalent volume of matching placebo. The placebo was identical to the azithromycin in appearance and taste. Dosage was determined by height stick approximation if the child was able to stand or by weight if the child was under 12 months of age and/or unable to stand [18]. The medication was administered as an oral suspension with a plastic dosing cup or syringe. All treatments were directly observed by the study team and recorded in the electronic mobile application.

### Randomization

The randomization sequence was generated by the study statistician without blocking or stratifying in R. Unique participant identification numbers were created which were associated with the randomization assignment and uploaded into the electronic data capture platform. The trial was double masked and all field team members and participants were masked. The allocation concealment mechanism was a combination of the unique participant IDs and matching drug labels. The drug was labeled with 1 of 8 different letters to avoid the possibility of unmasking. After a participant ID was assigned to the child, the field team member scanned the ID into the electronic mobile application which then informed the team member which letter to treat the child with.

### Follow-up assessment

Caregivers presented to the hospital 14 days after the baseline visit. A brief interview was conducted with the caregiver regarding any adverse events experienced by the child since treatment including abdominal pain, diarrhea, vomiting, constipation, or skin rash. These adverse events were specifically asked based on findings from previous pediatric azithromycin trials [19–21]. Caregivers also reported if healthcare was sought for the child since treatment and the diagnosis (e.g. diarrhea, pneumonia, malaria).

### Outcomes

The primary outcome for this trial was Shannon’s and Simpson’s diversity index of the gut microbiome at 6 months and will be reported separately. Secondary outcomes included malaria status at 14 days post enrollment determined by RDT, clinical malaria at 14 days post enrollment defined by a positive RDT and tympanic temperature ≥ 37.5 °C, caregiver-reported adverse events, and clinic visits.

### Sample size

The sample size was based on the primary outcome of the trial which was Shannon’s and Simpson’s diversity index of the gut microbiome. For the malaria outcome, we assumed 80% power to detect a significant effect with a sample size of 225 per arm, no loss to follow-up, and RDT positivity prevalence in the control group of 10%. Given these assumptions, the trial was powered to detect an absolute difference of 6.6%.

### Statistical methods

Descriptive baseline characteristics were summarized with proportions for categorical variables and medians and interquartile ranges (IQR) for continuous variables. The proportion of participants with a fever (defined as tympanic temperature ≥ 37.5 °C) at the time of the follow-up visit was calculated. We also calculated the proportion of children with a positive malaria RDT at the 14-day visit. Caregiver-reported health center visits were classified by arm and reason for the visit. Lastly, we calculated the proportion of children experiencing any adverse event as reported by their caregiver by study arm as well as each individual adverse event. Odds ratios (OR) and 95% confidence intervals (CI) were computed for each outcome using an unadjusted logistic regression model with the randomized treatment arm assigned as the predictor. Because RDTs can remain positive for several weeks even if there is no longer an active infection, we restricted the 14-day RDT model to children who were RDT negative at baseline as a sensitivity analysis. All analyses were intention-to-treat, where all randomized children were included regardless if they received their randomized assignment or not. Analyses were performed in Stata version 15.1 (StataCorp, College Station, TX).

## Results

**Fig. 2:**
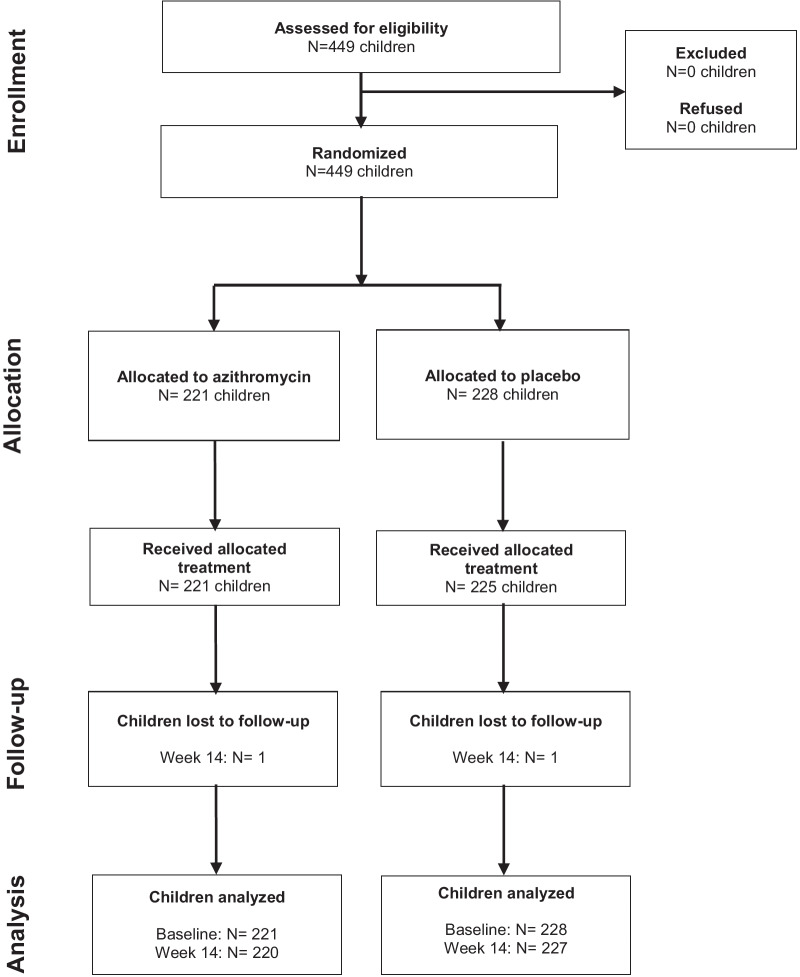
CONSORT flow diagram of study participants

A total of 449 children were enrolled in the trial with 221 in the azithromycin arm and 228 in the placebo arm (Fig. 2). Baseline characteristics were balanced between groups (Table 1). The median age was 32 months for the azithromycin group and 32.5 for the placebo group. Three participants were under 1 month of age with the youngest being 32 days old. In both groups, 48% were female. Among the 449 children enrolled, 446 (99%) received their study treatment. At baseline, 10% of children in the azithromycin group and 7% in the placebo group were RDT positive. Two percent of participants in the azithromycin arm and 0.4% of participants in the placebo arm had a positive malaria RDT plus fever. Two children (1 per arm) were lost to follow-up (Fig. 2).

**Table 1:** Baseline characteristics by treatment group

	Azithromycin (N = 221)	Placebo (N = 228)
Child’s age, months, median (IQR)	32 (21 to 44)	32.5 (22 to 44.5)
Female sex, N (%)	107 (48%)	110 (48%)
Currently breastfeeding, N (%)	64 (29%)	51 (22%)
Mother’s age, years, median (IQR)	26 (23 to 31)	27 (23 to 32)
Mother is literate, N (%)	92 (42%)	97 (43%)
Positive malaria RDT	22 (10%)	15 (7%)
Fever^a^ N (%)	23 (11%)	21 (9%)
Positive malaria RDT plus fever	4 (2%)	1 (0.4%)

*RDT* rapid diagnostic test, *IQR* interquartile range

^a^Fever defined as tympanic temperature ≥ 37.5 °C

We did not find any evidence of a difference between the azithromycin and placebo arms for malaria or clinical outcomes at 14 days (Table 2). Malaria RDT positivity remained similar in the placebo group (7%) and azithromycin group (8%) (OR 1.18 for azithromycin vs placebo, 95% CI 0.58 to 2.37, *P* = 0.65). At the 14-day follow-up, 33 children (15%) in the azithromycin group had a tympanic temperature ≥ 37.5 °C versus 47 children (21%) in the placebo group. Three percent of children in both arms had a positive malaria RDT plus fever at 14 days. There were 15 participants with a negative RDT at baseline which then became positive at 14-days (3% azithromycin; 4% placebo, OR: 0.90, 95% CI 0.32 to 2.45, *P* = 0.85). We did not find evidence of an effect of azithromycin on RDT positivity without fever (Table 2).

**Table 2:** Malaria and clinical outcomes at 14 days by treatment group

	Azithromycin N (%)	Placebo N (%)	Odds ratio (95% CI)	*P*-value
Positive malaria RDT	18 (8%)	16 (7%)	1.18 (0.58 to 2.37)	0.65
Fever^a^	33 (15%)	47 (21%)	0.68 (0.41 to 1.10)	0.12
Positive malaria RDT and fever	6 (3%)	6 (3%)	1.03 (0.33 to 3.25)	0.96
Positive malaria RDT without fever	12 (5%)	10 (4%)	1.25 (0.53 to 2.96)	0.61
Any health center visit	5 (2%)	9 (4%)	0.56 (0.19 to 1.71)	0.31
Reason for health center visit				
Malaria	0 (0%)	2 (1%)	N/A	N/A
Pneumonia	0 (0%)	0 (0%)	N/A	N/A
Diarrhea	3 (1%)	3 (1%)	1.03 (0.21 to 5.17)	0.97

*RDT* rapid diagnostic test, *CI* confidence interval

^a^Fever defined as tympanic temperature ≥ 37.5 °C

Caregivers of 3% of participants reported their child had a health center visit by the 14-day visit with no evidence of a difference between arms (2% azithromycin; 4% placebo, OR: 0.56, CI 0.19 to 1.71, *P* = 0.31). Diarrhea was the most common reason for a health center visit across both arms (OR 1.03 for azithromycin vs placebo, 95% CI 00.21 to 5.17, *P* = 0.97).

The results of the sensitivity analysis did not qualitatively change the results as we found no evidence of an effect of azithromycin on 14-day malaria RDT positivity in children with a negative RDT at baseline (OR 0.95 for azithromycin vs placebo, 95% CI 0.34 to 2.68, *P* = 0.93).

Overall, 12% of caregivers reported that their child experienced at least a single adverse event in the 14-day period after treatment with 20 in the azithromycin arm (9%) and 32 in the placebo arm (14%) (Table 3). Caregivers of children in the azithromycin arm had lower odds of reporting fever as an adverse event compared to children in the placebo group (OR 0.41, 95% CI 0.18 to 0.96, *P* = 0.04). Diarrhea was the most commonly reported adverse event and there was no significant difference between study arms (5% azithromycin; 8% placebo; OR 0.61, 95% CI 0.28 to 1.33, *P* = 0.21). We did not find evidence of a difference in any other adverse events.

**Table 3:** Adverse events at 14 days by treatment group

	Azithromycin N (%)	Placebo N (%)	Odds ratio (95% CI)	*P*-value
Any adverse event	20 (9%)	32 (14%)	0.61 (0.34 to 1.10)	0.10
Fever	8 (4%)	19 (8%)	0.41 (0.18 to 0.96)	0.04
Diarrhea	11 (5%)	18 (8%)	0.61 (0.28 to 1.33)	0.21
Vomiting	6 (3%)	14 (6%)	0.43 (0.16 to 1.13)	0.09
Abdominal pain	2 (1%)	4 (2%)	0.51 (0.09 to 2.82)	0.44
Constipation	1 (1%)	2 (1%)	0.51 (0.05 to 5.71)	0.59

*CI* confidence interval

## Discussion

We did not find evidence that a single oral dose of azithromycin reduces malaria positivity within a 14-day period after treatment. One consideration is the coinciding seasonal malaria chemoprophylaxis (SMC) that was administered at the same time as the trial within the Nouna community. SMC distributions with sulfadoxine–pyrimethamine (SP) and amodiaquine (AQ) to children aged 3–59 months occurred monthly from July 13^th^–16th, August 12th–15th, September 11th–14th, and October 10th–13th. A trial in Burkina Faso and Mali found SMC + AZ provided additional protection from malaria, but this effect was limited to the first 2 weeks post-administration [22]. Concomitant SMC distribution during the trial period may explain the lower than expected malaria prevalence we observed (8% RDT positivity at baseline and follow-up) [23–26].

Community-level distribution of AZ could be more effective for malaria control compared to individual-level distribution as several community based trials have demonstrated that mass AZ distribution reduces malaria parasitemia [8, 24, 27, 28]. For instance, a subset of villages in the MORDOR Niger trial reported that communities receiving azithromycin had half the odds of malaria parasitemia compared to communities treated with placebo [7, 8]. Trachoma trials have also documented a reduction in malaria parasitemia following mass azithromycin distribution, although the evidence is mixed [24, 26, 28–31]. Additionally, distributing AZ to communities may provide limited vector control as some studies suggest azithromycin decreases mosquito lifespan when ingested [32]. While individual-level interventions may have some impact on malaria transmission, it may be difficult to show a difference if the rate of reinfection is high, as during the peak transmission season. The present study did not collect serological data which could measure force of infection or measure the entomological inoculation rate. Community-level AZ interventions may have a greater impact on malaria transmission compared to individual-level interventions, but more research is needed.

We observed a lower probability of caregiver-reported fever in the azithromycin group compared to the placebo group, suggesting that azithromycin may have an effect on non-malaria fevers. Gastroenteritis and pneumonia were reduced 30% and 34% respectively in a West African azithromycin trial, suggesting AZ may lower other fever-inducing infections commonly found in sub Saharan Africa [22, 25]. There were no other significant differences in other adverse events or clinic visits. Administration of azithromycin to preschool aged children appears to be safe and well tolerated [19, 20].

The implications of utilizing an antibiotic, such as azithromycin, for malaria prophylaxis should be considered. Selection pressure for antimicrobial resistance can arise following over-administering antibiotics. Resistance to antibiotics could render treatment against other bacterial infections ineffective, which is a serious concern for global health [33]. The results of this study do not suggest that the use of azithromycin for prevention or treatment of malaria at the individual level is appropriate, and given concerns for selection for resistance, its use should be avoided.

Limitations for this study include the sole use of an RDT rather than PCR or microscopy for defining malaria positivity. Some trials have documented low RDT sensitivity in this setting with results that may vary by parasite density [34, 35]. RDTs generally perform worse with low parasite density infections, but due to the randomized nature of the study we do not expect any differential bias between study arms [36]. Future studies might consider performing more than one RDT test per individual where PCR capabilities are limited. Other limitations should be taken into consideration. Because the trial was designed and powered for a microbiome primary outcome, the trial was likely underpowered to detect differences for malaria specific outcomes. Azithromycin is rapidly absorbed and has a long half-life, but the 2-week duration of the study may have been too short to demonstrate significant effects [37]. Additionally, we did not collect data regarding which children in the trial specifically received SMC for prevention or artemisinin-based combination therapy for those with a malaria diagnosis. While concomitant SMC distribution may reduce the prevalence of malaria parasitemia and reduce power, due to the randomized nature of the study it would not affect inferences. However, effects may be closer to the null than in the absence of SMC. Lastly, this study took place in a single peri-urban town that may not be generalizable to other communities. Nouna Town residents have access to health clinics and may have better health outcomes compared to more rural communities with different malaria transmission patterns. Future research should aim to select a larger number of communities that are not exclusively urban areas.

## Conclusion

Azithromycin did not lower malaria positivity as measured by RDT within a 14-day period when administered as a single dose to children 1–59 months old. The point prevalence of fever was similar between the azithromycin and placebo groups, but caregiver-reported fever over the 14-day period from treatment occurred less often among children receiving azithromycin compared to placebo. We found no evidence that individual-level treatment with azithromycin affected malaria prevalence 2 weeks after treatment.

## Data Availability

All data is publicly available on OSF (www.osf.io).

## Abbreviations

AQ: Amodiaquine
AZ: Azithromycin
CRSN: Centre de Recherche en Santé de Nouna
CI: Confidence interval
HDSS: Health and Demographic Surveillance System
MDA: Mass Drug Administration
OR: Odds ratio
SMC: Seasonal malaria chemoprevention
SP: Sulfadoxine–pyrimethamine
RDT: Rapid diagnostic test
USA: United States of America
